# Supplementary material for: Picture perfect science communication: How public audiences respond to informational labels in cinematic-style 3D data visualization
Source: PLoS One. 2024 Oct 17;19(10):e0307733. doi: 10.1371/journal.pone.0307733 (PMC11486383; doi:10.1371/journal.pone.0307733)
Supplement: Attachment — Submitted filename: renamed_4d0a9.docx [file pone.0307733.s001.docx]

**Response to Reviewers – Oct. 2023**

While I don’t agree with all the feedback, I am very appreciative to this reviewer for taking the time to give detailed comments. I believe the manuscript has already improved because of this feedback.

| **Reviewer comment** | **Author response** |
| --- | --- |
| 1. While studies don't need to be complicated to be valuable, your study seems too simple (i.e. this is a single factorial design with two conditions). Many studies have examined data visualization in science communication. I would suggest one or more of the following to fix this:  a. add in another variable, b. provide further reasoning for your paper's ability to move science communication in data visualization literature forward, or c. mention your study's simplicity as a limitation in your limitations section  *Also, you could argue that your are adding to existing, similar lit related to data visualization in science communication as a form of replication to enhance this research area in science communication. | 1. The reviewer consistently refers to ‘data visualization’ in general as the focus of the paper. In fact, the paper clearly states that it is focused on a more specific category: 3D cinematic-style data visualization aimed at the general public. This particular type of data visualization has not in fact been studied many times as the reviewer contends. In fact, this is the first study addressing it empirically (here is a previously published peer reviewed literature review that addresses this gap: https://www.frontiersin.org/articles/10.3389/fcomm.2022.840631/full). 2. I don’t agree that a focus on ‘treatment’ and ‘control’ conditions in a randomized survey experiment constitutes a methodological limitation. ‘Simple’ has a pejorative connotation to me. So I have added a reference to the design being ‘straightforward’ as a synonym in both the Methods section and the Discussion. |
| 2. While you mention what you are doing, there are no formally proposed hypotheses or research questions in this paper. These are important to have, because they provide structure for the rest of your paper.  Your lack of research questions and hypotheses make your results section confusing in terms of what you are trying to specifically accomplish with your collected data in this paper (aside from your overarching question on your audience’s perceptions of videos with or without labels).  Hypotheses can straighten out your paper for you and the reader pertaining to what variables you are measuring, hint at what statistical analyses you will be using, and whether your findings were significant or not. It makes writing your discussion a lot easier too. | I have now re-framed the stated study aim to be in the form of a research question, and added some clarifications about what is being measured. |
| 3. On pg. 6, in the last paragraph of your literature review you mentioned entities like level of immersion yet there's no section in your literature review that covers this topic. Level of immersion is an important concept to address and elaborate on for papers covering anything with visual technology or media. | I have extended the discussion on this topic in the literature review. |
| 4. There is no formally introduced theoretical framework in this paper. Is it the hierarchy of cinematic scientific visualization needs? If so, give it its own section and elaborate on how this theory pertains to your study in your lit review. Otherwise, there are many theoretical frameworks (social and psychological) that would work well with this article’s topic. | Formally introduced theoretical frameworks are not required in PLOS ONE author guidelines. |
| 5. At the beginning of the method section, you mentioned that this is an experiment, but then you don't include some specificities involved in an experimental design. For example, your factorial design is not listed (it would be a single factorial design with 2 conditions for this study).  You mentioned conditions, but you are not concrete enough about what independent variables are being manipulated within each condition. Explaining these things and including a diagram of the variables you're manipulating, your mediating and moderating variables, and your outcome variables would be really helpful to those who are and are not familiar with your research. | I have now made the IVs and DVs explicit in the methods section (using this terminology). |
| 6. Your pilot study explanation is not clear enough. You need to explain what these adjustments were to your labels. You should add-in statistics substantiating the validity of your examined variables in your pilot to enhance the validity of your manipulations in your main study as well. | As indicated in the manuscript, this is a whole published study in itself (already published in another open-access journal). It would be a waste of space in this article to describe the adjustments.  The request for statistics to validate the variables comes from a psychometric measurement tradition that I do not subscribe to. |
| 7. For your collected sample, you mention fully completed and partially completed responses. I had to pull out a calculator to figure out how you got 577 participants, because you don't explain whether you kept the partially completed responses or deleted them. You need to clearly explain what respondents you used for your data analysis. | This is specified explicitly now. |
| 8. Your instrument section should be at the beginning of the method section, not the end. | Moved up to right after ‘research design’ now. |
| 9. Your instrument section just states “finally, they answered a series of attitudinal and learning-related questions to measure their level of understanding of key content covered in the film” – you need to state what each of these measures are and a citation for each one on where you got the measure from. | These details were a bit later in the instrument section and also in the results section to avoid repetition. These details have now been centralized in the Instrument section in line with the reviewer’s suggestion.  The measures were created by the lead author for this study. There is no citation for each one. |
| 10. You need a section in your methods section that explain every outcome variable, control variable, and attention checking question that you measured in your survey. Each measure listed in that section should have a definition of the measure, an in-text citation for the source got the measure from, the items in each measure, whether the measure was a Likert or semantic differential scale, the number of items in the measure, and the Cronbach's alpha level. Each Cronbach's alpha should be above .7 and have more than three items to be considered a reliable measure. If it's below, provide rationalization for why you are still using the measure. | This is describing a psychometric approach to measurement. I don’t subscribe to this approach.  The Instrument section already indicates the type of measure (e.g., semantic differential). |
| 11. There is no power analysis or reasoning shown to establish why you chose to collect a sample size of n=577 participants. Without sample size reasoning, you have no way of knowing if your results are due to chance or are genuine.  A power analysis establishes an acceptable probability of making a type 1 or type 2 error for rejecting or failing to reject your null hypothesis for your experiment’s given circumstances. | The Methods section explains at length how the recruitment was conducted (it was open, not a purchase of a fixed sample number)  Posthoc power analysis results have now been added, showing the study was well-powered. |
| Minor issues  1. There are statements in this paper that need in-text citations for support. For example, on pg. 3, the statement “One promising science communication approach that has gained increasing attention in recent years is cinematic-style 3D data visualizations” needs citations. There are parts throughout the lit review that need evidence either through a citation or your own personal reasoning. | Citation note added |
| 2. Add in a model with all of your study's variables, and add in more tables for your data results (like your participant demographics). Then, just have the reader refer to them. | I don’t think that doing this will improve the paper. |
| 3. For your methods, you really only need the following sections: an intro after the Methods section title stating something like: "Study one was conducted using a single (with two levels: label and no label), between-subjects, factorial design via an online experiment." Then a Participants (talk about how you attained your sample, sampling method, sample size, power analysis, and reasoning as to way your sample and sampling method are appropriate for your study based on your variables used), Procedure and Stimulus, Measures, and Analytical Approach section. | I have integrated the suggested summary of the study design into the abstract. I don’t think changing the headings within the Methods section will make it clearer for the reader. |
