# Supplementary material for: Picture perfect science communication: How public audiences respond to informational labels in cinematic-style 3D data visualization
Source: PLoS One. 2024 Oct 17;19(10):e0307733. doi: 10.1371/journal.pone.0307733 (PMC11486383; doi:10.1371/journal.pone.0307733)
Supplement: Attachment — Submitted filename: renamed_47eaf.docx [file pone.0307733.s002.docx]

**Response to Reviewers (Rebuttal Letter) – Dec. 2023**

As previously noted, I am very appreciative to this reviewer for taking the time to give detailed comments and to follow up with additional suggestions. The manuscript has definitely improved because of this feedback.

| **Reviewer comment** | **Author response** |
| --- | --- |
| 1. You provide some great information and reasoning in your discussion section; however, your discussion section should dive into the “why” for your results a little more deeply. While many of your results were not significant (and you are of course not supposed to make inferences on non-significant results), you can still provide thoughtful reasoning as to why you think a relationship may not have occurred. For example, on page 22, you state:  “The three items used to evaluate the immersive nature of the videos – ease of mind, attention focus, and feeling as if in space – showed no statistically significant differences between the labeled and unlabeled videos.”  You could provide a sentence or two for each one (ease of mind, attention focus, and feeling as if in space) explaining why you think there was not a relationship between the variables that were tested. You can talk about the implications of these non-significant findings for this article’s area of research more deeply as well.  Many readers go straight to the discussion section of a research paper when looking for research to cite so this can help increase the number of times your article gets cited in future research. | **We really appreciate this suggestion. We have now added a few sentences accordingly.** |
| Minor Edits:  1. There is an instance on page 18 in the second paragraph of that page where an in-text citation reads “Error! Reference source not found.” Just make sure instances like these are fixed in the manuscript or provide reasoning for why these are written this way. | **Fixed** |
| 2. On page 22, in the first paragraph of the Discussion section, add a “)” after 574 in this sentence: “A mean score of 80.74 out of 100 represented a generally positive sentiment about video quality (n=574.” Again, while this manuscript is well-written, just make sure any little grammatical instances like this one are corrected throughout the manuscript. | **Fixed** |
| 3. On page 23, there is a link to a blog post embedded in this sentence: “To do this, we believe in taking an evidence-based communication approach, with audience research in a project to systematically test different design options.” Make sure to remove instances like this throughout the manuscript. | **Fixed** |
| 4. Some of the tables go off the page. This may be fixed when the manuscript is formatted after being accepted for publication; however, you could adjust the dimensions of these to fit the page. | **This will probably need to be addressed post acceptance alongside other formatting changes** |
